# Lipoaspiration for the Treatment of Lower Limb Lymphedema: A Comprehensive Systematic Review

**DOI:** 10.7759/cureus.5913

**Published:** 2019-10-15

**Authors:** Antonio J Forte, Maria T Huayllani, Daniel Boczar, Pedro Ciudad, Sarah A McLaughlin

**Affiliations:** 1 Plastic Surgery, Mayo Clinic Florida - Robert D. and Patricia E. Kern Center for the Science of Health Care Delivery, Jacksonville, USA; 2 Plastic, Reconstructive and Burn Surgery, Arzobispo Loayza National Hospital, Lima, PER; 3 Surgery, Mayo Clinic Florida - Robert D. and Patricia E. Kern Center for the Science of Health Care Delivery, Jacksonville, USA

**Keywords:** lipoaspiration, lymphedema, surgery, plastic surgery, chronic lymphedema, liposuction, lower extremity, treatment, reconstruction

## Abstract

Lipoaspiration is a potential treatment for lymphedema; however, there is a lack of knowledge regarding the outcomes and benefits of this procedure in lower limb lymphedema. We aim to describe the outcomes of studies to date reporting the use of lipoaspiration in lower limb lymphedema. We searched the PubMed database for studies that evaluated the use of lipoaspiration for lower limb lymphedema. The keywords “lipoaspiration” AND “lymphedema,” synonyms, and different combinations were used for the search. Only English studies were included. Eight studies met the inclusion criteria from a total of 129 articles. A volume reduction greater than 50% was found in all patients who underwent lipoaspiration for lower limb lymphedema. Complete volume reduction was found after four to five years of follow-up. A greater volume reduction was found for secondary lymphedema when compared to primary lymphedema. Finally, improvement was found in functionality, quality of life, and rate of infection. Lipoaspiration is recommended for patients with lower limb lymphedema in stages 2 and 3 of the disease, followed by controlled compressive therapy that maintains the volume reduction accomplished by the procedure.

## Introduction and background

Almost three million people in the United States and 90 million people worldwide have lymphedema [1-2]. Lower limb lymphedema accounts for 20% of all cases of lymphedema [3]. This condition is most likely caused by lymphadenectomy during the surgical treatment and radiotherapy of malignancies affecting the genitourinary tract and lower limb melanomas [3-4]. There are two types of lymphedema: the primary type, which is a result of the obstruction, malformation, or hypoplasia of the lymphatic channels or nodes and the secondary type, caused by obstruction or destruction of the well-developed lymphatic channels or nodes [5]. Regardless of the etiology, different surgical procedures are indicated for each case. Physiological procedures include the surgeries that attempt to reestablish lymphatic flow and include lymphavenous anastomosis and lymph node transfer [6]. Conversely, excisional procedures are indicated to remove the overgrown tissue in chronic lower limb lymphedema. Lipoaspiration offers better volume reduction with minimal morbidity when chronic lymphedema has resulted in fibroadipose accumulations and hypertrophy in the lower extremity [7]. However, to our knowledge, no studies have analyzed the outcome of this procedure in lower limb lymphedema.

The purpose of this study was to summarize and describe the studies to date that report lower limb lymphedema treated with lipoaspiration; in addition, this study also presents an overview of the surgical technique, postoperative care, and volume measurement techniques to evaluate improvement following this procedure and report the efficacy of lipoaspiration for lower limb lymphedema.

## Review

Methods

Study Selection

Our systematic review included all studies that evaluated the efficacy of liposuction in patients with lower limb lymphedema following the PRISMA guidelines. Studies were included if they reported volume measurements and changes after liposuction of the lower limb lymphedema and were written in English. Studies were excluded if they did not report postoperative volume changes, analyzed liposuction in combination with any other surgical treatment, or reviews.

Data Sources and Search Strategy

A comprehensive systematic review was conducted by one author (M.T.H.) on July 23, 2019 in the PubMed database for articles reporting on liposuction for treatment of lower limb lymphedema. A search strategy was generated using the following terms: ((((((((((((((((((Lipectomies[Title/Abstract]) OR Aspiration Lipectomy[Title/Abstract]) OR Aspiration Lipectomies[Title/Abstract]) OR Lipectomies, Aspiration[Title/Abstract]) OR Lipectomy, Aspiration[Title/Abstract]) OR Aspiration Lipolysis[Title/Abstract]) OR Lipolysis, Aspiration[Title/Abstract]) OR Suction Lipectomy[Title/Abstract]) OR Lipectomies, Suction[Title/Abstract]) OR Lipectomy, Suction[Title/Abstract]) OR Suction Lipectomies[Title/Abstract]) OR Lipolysis, Suction[Title/Abstract]) OR Suction Lipolysis[Title/Abstract]) OR Liposuction[Title/Abstract]) OR Liposuctions[Title/Abstract]) OR Lipoplasty[Title/Abstract]) OR Lipoplasties[Title/Abstract]) AND ((lymphedema[Title/Abstract]) OR lymphoedema[Title/Abstract]). Identified studies were uploaded into EndNote (Clarivate). Two independent reviewers (M.T.H. and D.B.) selected the final studies to report. One author (M.T.H.) manually screened the manuscripts and selected them according to the inclusion and exclusion criteria in a two-step process. First, studies were reviewed based on title and abstract. Second, the full text of the selected studies was screened for the final decision. If there was any doubt in the selection of an article, a second author (D.B.) reviewed the manuscripts and according to the selection criteria, both reviewers came to an agreement for the final selection.

Data Pooling and Data Analysis

The main data were extracted and pooled. The variables selected to describe the studies to date included author, year of publication, type of study, cause of lymphedema, number of patients, age, International Society of Lymphology stage, duration of lymphedema, lipoaspirate volume, measurement tool, follow-up, and outcomes.

Results

**Figure 1:**
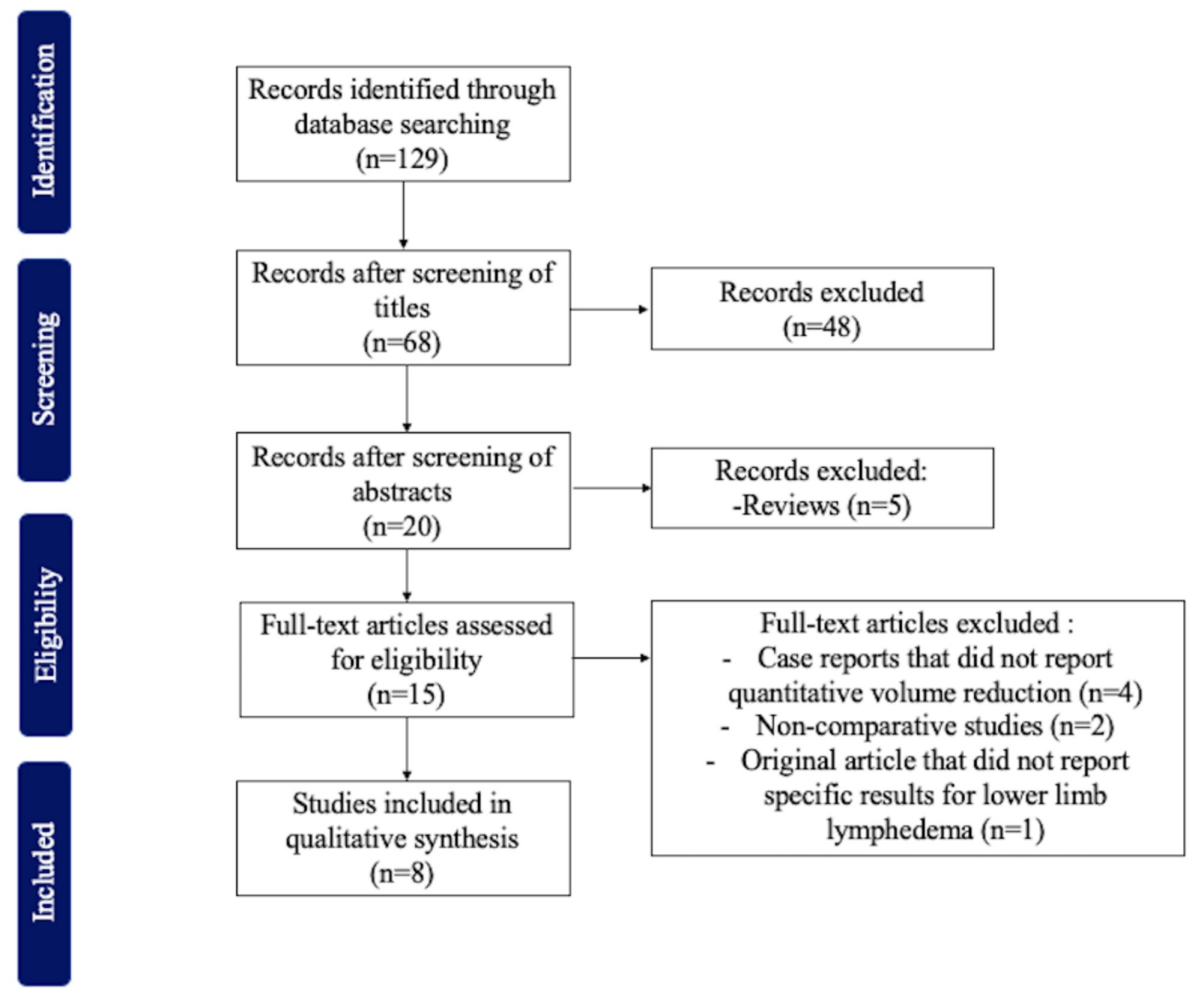
Inclusion and exclusion criteria

In our first search, 129 articles were found. From these, only eight studies met the inclusion criteria (Figure 1). All included studies were published between 2006 and 2018 (Table 1). All the studies considered patients treated with liposuction after conservative compression therapy. A total of 191 patients, 95 with primary and 96 with secondary lymphedema, were treated with lipoaspiration; at the time of reporting, most were in ISL stage 2 or 3 [8-11]. The mean duration for lymphedema ranged between 10 and 19 years, and the maximum lipoaspirate volume extracted reached 5,300 mL [8-9,11]. Five studies compared preoperative and postoperative volumes of affected limbs using the 4-cm truncated cone circumferential method [8-10,12-13]. One study used the optoelectronic volume-measuring device; one, bioimpedance; one, the disc model method; and one, plethysmography [10-11,14-15]. In general, edema was considerably reduced in the affected limb in all patients who underwent lipoaspiration followed by controlled compression therapy for lower limb lymphedema. However, the complete reduction was mainly observed after four to five years of treatment and follow-up [8-9,15]. A greater volume reduction was found in patients with secondary lymphedema compared to primary lymphedema [8,14]. Additionally, changes in quality of life and function were also evaluated in one study, reporting improvement after surgery [9].

**Table 1:** Published studies to date reporting outcomes of liposuction to treat lower limb lymphedema R, retrospective; P, prospective; CR, case report; NS, not specified; LS, liposuction; ISL, International Society of Lymphology; PSFS, patient-specific functional scale [8-15]

Author	Year	Type of study	Cause	Number of patients	Age	ISL Stage	Duration of lymphedema (mean in years)	Lipoaspirate volume (ml)	Measurement Tool	Follow-up (months)	Outcomes
Stewart CJ et al.	2018	P	Primary	42	Mean: 46	2, 3	19	Mean: 4,550	4 cm truncated cone circumferential measurement	96	Volume reduction
			Secondary	30							
McGee P et al.	2018	P	Secondary	21	Mean: 52	2,3	15.2	Mean: 5,300	4 cm truncated cone circumferential measurement, Quality of Life Inventory (LyQli)	60	Volume reduction, better quality of life
Lamprou DA et al.	2017	P	Primary	47	Mean: 43.6	NS	20	Median: 3,750	Optoelectronic volume measuring device	24	Volume reduction, incidence of cellulitis decreased
			Secondary	41	Mean: 52	NS	12	Median: 3,975			
Lee M et al.	2016	CR	Primary	1	65	NS	NS	NS	4 cm truncated cone circumferential measurement	15	Volume reduction
Boyages J et al.	2015	p	Primary	2	Mean: 50.7	2,3	15.5	NS	4 cm truncated cone circumferential measurement, bioimpedance, PSFS	18	Volume reduction, L-Dex measures reduction, an improvement in the PSFS index
			Secondary	4							
Espinosa-de-Los-Monteros et al.	2009	CR	Primary	1	26	3	10	Right leg: 1,650 Left leg: 1,250	Disc model method	14	Volume reduction
Brorson H et al.	2008	CR	Primary	1	27	NS	16	3,430	Plethysmography	48	Volume reduction
Greene AK et al.	2006	CR	Primary	1	34	NS	10	Right leg: 2,300 Left leg: 2,000	4 cm truncated cone circumferential measurement	18	Volume reduction

Indications

Lipoaspiration for the lower extremity lymphedema should be indicated for ISL stage 2 or 3 of disease independent of the cause of origin in patients with chronic symptoms. Moreover, conservative compressive therapy should be offered first; if no additional improvement is seen in the reduction of lower limb volume, surgical treatment may be considered.

Surgical Technique

Lipoaspiration should be performed under general anesthesia and followed by limb exsanguination and tourniquet application [10]. The procedure should start with the infiltration of 1,000 mL of Ringer lactate solution with 250 mg of lidocaine and 1 mg of epinephrine 1:1,000, or with the tumescent technique with 20 mL of 0.5% levobupivacaine and 1 mg of 1:1,000 adrenaline in 1 L of Hartmann solution in the subcutaneous tissue of the ankle and dorsum of the affected leg for allowing vasoconstriction [8,11]. Then, using cannulas of 22 and 30 cm in length and 3 to 5 mm wide [8, 14], a circumferential suctioning of all the fat should be performed through 12 incisions and a vacuum pump, from foot to thigh, until tissue consistency is soft and smooth. When the procedure is finished, the incisions should be left open to allow drainage of fluid and bandages should be applied [9].

Postoperative Care

Intravenous antibiotics should be given in the first 48 hours and changed to oral administration for seven days more after surgery [9]. Moreover, thromboembolism prophylaxis can be given with 2,500 units of dalteparin [8]. Postoperative pressure should be applied continuously with pressure bandaging under controlled compression therapy, in which custom-made compression garments of 50 to 80 mmHg are applied to reduce swelling after surgery [10,15]. These compression garments should be used continuously and changed regularly approximately three to four times in the first year as the volume decreases. Volumetric measurements will need to be performed at one, six, 12, and 24 months of follow-up to evaluate improvement. However, their constant use will probably maintain the results over time.

Volume Measurements

The most common technique to measure the lower limb volume of lymphedema was the 4 cm truncated cone circumferential measurement. This technique determined different volume segments using a formula of the truncated cone, where the circumference of the proximal and distal limb limit the cone and adding all volumes to calculate the total volume of the limb [16]. Another method used to measure the lower limb volume was the optoelectronic volume measuring device, which is a perometer compound of a square frame that can calculate the volume, circumference, contour, and cross-sectional area through an infrared light emitted to the lower limb and detected when moved along each leg. On the other hand, the bioelectrical impedance analysis method evaluates extracellular fluid using a low-voltage electrical current: When lymphedema is present, the extracellular fluid volume increases and, as a consequence, the current flow decreases [17]​​​​​​​. The L-Dex ratio is the bioimpedance unit calculated from the impedance of the unaffected limb after a low-frequency current divided by the affected limb. The third method used to measure lower limb lymphedema was the disc model method, which obtained the volume through an equation that uses the circumferences of the limbs measured each 40 mm from the proximal to the distal part of the limb [18]. Plethysmography, the last tool used, the measured volume inside water- or air-filled chamber and was able to detect subtle changes in the volume of the limb over time [15].

Discussion

Lower limb lymphedema is a condition that can be treated using several different surgical options. Lipoaspiration is one of the surgical procedures that offer a small incision with the possibility of good recovery in many of these patients; however, few reports have been done that examine outcomes following this procedure [19]. The present review aimed to evaluate whether lipoaspiration is efficient for the treatment of lower limb lymphedema.

The anatomic characteristics of the lower limb (e.g., a thin subcutaneous fat layer and a heterogeneous distribution of fat, depending on the location in the leg) have been thought to influence the results of lipoaspiration in lower extremity and suggest an increased risk of poor satisfaction, abnormal pigmentation, border irregularity, ulceration, and persistent edema [20]. However, our systematic review found that all studies reported volume reduction following lipoaspiration. Many studies of lower limb liposuction for lymphedema demonstrated positive outcomes, especially for patients with late stages of lymphedema where the lymph vessels were completely absent [11]. A case report in 2006 described a patient with lower limb lymphedema who experienced a 75% reduction in preoperative volume 18 months post surgery, with none of the aforementioned possible complications [13]. In another case, a volume reduction of 71% was reached after 12 months of lipoaspiration, although the volume was unchanged until 18 months, then continued decreasing to 75% at two years of follow-up, with complete reduction four years post lipoaspiration [15]. Another review of six patients with lower limb lymphedema revealed complete volume reduction after 12 months of follow-up, and an isolated case described an excess volume reduction of 86% at 15 months after surgery [10,12]. On the other hand, one study reported different volume reductions according to the cause of lymphedema: At 24 months post lipoaspiration, a reduction of 79% was achieved in patients with primary lymphedema, and a volume reduction of 101% was found in patients with secondary lymphedema [14]. A possible explanation of this difference between primary and secondary lymphedema may be attributed to the shorter duration of lymphedema in patients with secondary lymphedema compared to those with primary lymphedema. Moreover, patients with secondary lymphedema might have had more content of water which could have reduced by the controlled compressive therapy after the lipoaspiration. The difference between primary versus the secondary cause of lymphedema was also found by Stewart et al [8]. They explained that lower volume reduction was shown in primary cases due to the absence of lymphatic vessels as known by the pathogenesis and that many of these patients present with greater volumes around the knee compared to the normal leg that makes volume reduction more difficult [8]. In general, an interesting finding of our review was the fact that a complete reduction was found only after four to five years of surgical treatment. The reason for the delay in the reduction of the lower limb volume may be related to gravity, which makes it more difficult to decrease the volume in the affected lower limb through controlled compressive therapy after surgery. Moreover, the final outcomes also depend on patient compliance regarding the use of compression garments after lipoaspiration.

In addition to the benefits in volume reduction reported for lipoaspiration of lower limb lymphedema, an improvement in the functionality, quality of life, and a decrease in the rate of infections were found after lipoaspiration of lower limb lymphedema. Boyages et al. reported an improvement in the functional score using the patient-specific functional scale (PSFS) specifically in pain, heaviness, self-consciousness, anxiety, perceived swelling, and emotional impact [10]. Moreover, an increase in lower limb mobility, activities of daily living, better quality of life, and absence of infection was found after the procedure [12]. Lamprou et al. reported a decrease in the rate of infection reaching 0.2 and 0.3 attacks per year for primary and secondary lymphedema, respectively [14].

As a result of all reported studies, lipoaspiration is recommended for patients with ISL stage 2 or 3 in the reduction of fat and fibrosis presented in the late stages of the disease.

Strengths and Limitations

Limitations of our study include the presence of heterogeneity between studies due to the different techniques of volume measurements, follow-up, and protocol of the procedure established in each study, which made comparisons of statistical analyses among studies difficult. In addition, inherent limitations of a review methodology may carry out to search, selection and publication biases that should be considered. However, we believe these reported data are valuable as this study is, to our knowledge, the first systematic review to analyze the outcomes of this technique for the treatment of lower limb lymphedema.​​​​​​​

## Conclusions

Lipoaspiration for treatment of lower limb lymphedema, followed by controlled compressive therapy (which must be continued to maintain the reduction volume reached by lipoaspiration), can potentially improve late stages 2 and 3 of chronic lymphedema when conservative therapy is ineffective. This procedure has been demonstrated to improve functionality and quality of life and decrease the rate of infection in lower limb lymphedema.

## References

[1] Carl HM, Walia G, Bello R (2017). Systematic review of the surgical treatment of extremity lymphedema. J Reconstr Microsurg.

[2] Garza R 3rd, Skoracki R, Hock K, Povoski SP (2017). A comprehensive overview on the surgical management of secondary lymphedema of the upper and lower extremities related to prior oncologic therapies. BMC Cancer.

[3] Cormier JN, Askew RL, Mungovan KS, Xing Y, Ross MI, Armer JM (2010). Lymphedema beyond breast cancer: a systematic review and meta-analysis of cancer-related secondary lymphedema. Cancer.

[4] Bakar Y, Tugral A (2017). Lower extremity lymphedema management after gynecologic cancer surgery: a review of current management strategies. Ann Vasc Surg.

[5] Ciudad P, Sabbagh MD, Agko M (2019). Surgical management of lower extremity lymphedema: a comprehensive review. Indian J Plast Surg.

[6] Greene AK, Maclellan RA (2016). Operative treatment of lymphedema using suction-assisted lipectomy. Ann Plast Surg.

[7] Schaverien MV, Coroneos CJ (2019). Surgical treatment of lymphedema. Plast Reconstr Surg.

[8] Stewart CJ, Munnoch DA (2018). Liposuction as an effective treatment for lower extremity lymphoedema: a single surgeon's experience over nine years. J Plast Reconstr Aesthet Surg.

[9] McGee P, Munnoch DA (2018). Treatment of gynaecological cancer related lower limb lymphoedema with liposuction. Gynecol Oncol.

[10] Boyages J, Kastanias K, Koelmeyer LA (2015). Liposuction for advanced lymphedema: a multidisciplinary approach for complete reduction of arm and leg swelling. Ann Surg Oncol.

[11] Espinosa-de-Los-Monteros A, Hinojosa CA, Abarca L, Iglesias M (2009). Compression therapy and liposuction of lower legs for bilateral hereditary primary lymphedema praecox. J Vasc Surg.

[12] Lee M, Perry L, Granzow J (2016). Suction assisted protein lipectomy (SAPL) even for the treatment of chronic fibrotic and scarified lower extremity lymphedema. Lymphology.

[13] Greene AK, Slavin SA, Borud L (2006). Treatment of lower extremity lymphedema with suction-assisted lipectomy. Plast Reconstr Surg.

[14] Lamprou DA, Voesten HG, Damstra RJ, Wikkeling OR (2017). Circumferential suction-assisted lipectomy in the treatment of primary and secondary end-stage lymphoedema of the leg.. Br J Surg.

[15] Brorson H, Ohlin K, Olsson G, Svensson B, Svensson H (2008). Controlled compression and liposuction treatment for lower extremity lymphedema. Lymphology.

[16] Brorson H, Hoijer P (2012). Standardised measurements used to order compression garments can be used to calculate arm volumes to evaluate lymphoedema treatment. J Plast Surg Hand Surg.

[17] Fu MR, Cleland CM, Guth AA (2013). L-Dex ratio in detecting breast cancer-related lymphedema: reliability, sensitivity, and specificity. Lymphology.

[18] Chromy A, Zalud L, Dobsak P, Suskevic I, Mrkvicova V (2015). Limb volume measurements: comparison of accuracy and decisive parameters of the most used present methods. Springerplus.

[19] Qi F, Yang Y, Gu J, Shi Y (2009). Long-term follow-up of the treatment of lower limb lymphedema with liposuction. Plast Reconstr Surg.

[20] Weniger FG, Calvert JW, Newton ED (2004). Liposuction of the legs and ankles: a review of the literature. Plast Reconstr Surg.

